# Bromisoval-induced bromism with status epilepticus mimicking Wernicke’s encephalopathy: report of two cases

**DOI:** 10.1186/s12883-022-02712-3

**Published:** 2022-05-16

**Authors:** Masahiro Biyajima, Shunichi Satoh, Takahiro Morikawa, Yuki Morita, Rie Watanabe, Daisuke Matsui, Masataka Konno, Nobutoshi Morimoto, Yuichi Yatsu, Akihito Hirasaki, Hiroyuki Yahikozawa

**Affiliations:** 1 Department of Neurology, Nagano Red Cross Hospital, 5-22-1, Wakasato, Nagano, 380-8582 Japan; 2 Department of Nephrology, Nagano Red Cross Hospital, Nagano, Japan; 3 Department of Psychiatry, Nagano Red Cross Hospital, Nagano, Japan; 4 Department of Neurology, Kagawa Prefectural Central Hospital, Takamatsu, Kagawa Japan; 5 Department of Anesthesiology, Kagawa Prefectural Central Hospital, Takamatsu, Kagawa Japan

**Keywords:** Bromism, Bromisoval, Bromovalerylurea, Status epilepticus, Wernicke’s encephalopathy, Korsakoff’s syndrome

## Abstract

**Background:**

Bromine compounds are used in several drugs, including over-the-counter drugs. They sometimes cause intoxication known as bromism. Although the acute neurological symptoms and sequelae of bromism vary, few reports have mentioned acute encephalopathy.

**Case presentation:**

We report two cases of bromisoval-induced bromism with status epilepticus. Presence of pseudohyperchloremia and history of over-the-counter medication use guided the diagnosis. In the acute phase, our patients showed bilateral medial thalamic lesions on magnetic resonance imaging. The imaging findings were similar to those of Wernicke’s encephalopathy. Although these findings improved in the chronic phase, neuropsychiatric sequelae, such as confabulation and amnesia, occurred.

**Conclusion:**

Bromism can cause acute encephalopathy, and it is important to differentiate it from Wernicke–Korsakoff syndrome.

## Background

Bromisoval (bromovalerylurea, BVU) was discovered in 1907. It is used as a sedative and hypnotic. However, its sale has been banned in most countries owing to the risks of addiction and suicide, and safer drugs, such as benzodiazepines, have been developed. Nevertheless, BVU, a component of antipyretic analgesics, is still easily available in Japanese drugstores. BVU intoxication, also known as bromism, causes various symptoms, including neurological symptoms such as ataxia, dysarthria, and gait disturbance; psychiatric symptoms such as anxiety and confusion; dermatological symptoms (bromoderma); and gastrointestinal symptoms. Bromism is caused by bromide intoxication following exposure to inorganic (potassium bromide) and organic (BVU and methyl bromide) bromine compounds. Many reports of methyl-bromide-induced bromism exist, since it is widely used for fumigation [1]. Dextromethorphan-hydrobromide-induced bromide intoxication has been reported in the United States [2]. BVU intoxication has been reported in Japan and Taiwan [3, 4]. Bromism is often difficult to diagnose owing to its various pathological forms and the need for the history of over-the-counter drug use [5].

We report two cases of bromism with status epilepticus and bilateral medial thalamic lesions on magnetic resonance imaging (MRI). The MRI findings and neuropsychiatric symptoms were similar to those associated with Wernicke’s encephalopathy (WE) and Korsakoff’s syndrome (KS), an acute encephalopathy and its complications caused by thiamine deficiency. The presence of marked hyperchloremia and findings from detailed history-taking regarding the drug use resulted in the diagnosis of BVU intoxication. It is important to consider bromism in patients with unexplained disturbance of consciousness, epileptic seizures, and hyperchloremia; moreover, differentiating between WE and bromism is crucial owing to the similarities in the symptoms and imaging findings of these two conditions.

## Case presentation

### Case 1

**Fig. 1:**
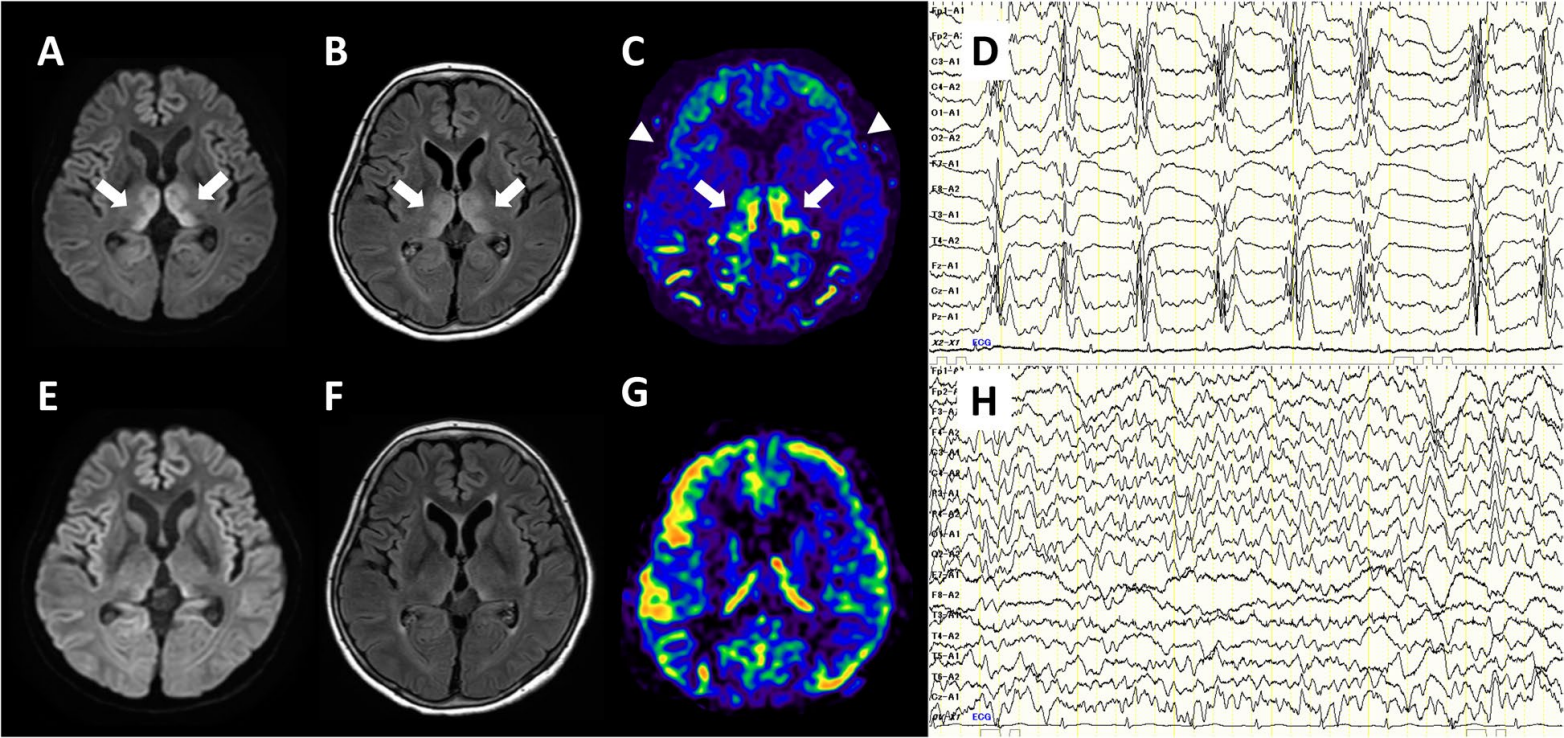
Brain magnetic resonance imaging and electroencephalography findings of Patient 1. Magnetic resonance image (**A**: diffusion-weighted imaging, **B**: fluid-attenuated inversion recovery imaging, **C**: Arterial spin labeling perfusion imaging) obtained in the acute phase shows high-intensity lesions and increased blood flow in the medial thalamus (arrows) and decreased blood flow in the cerebral cortex (arrowheads). Electroencephalogram obtained in the acute phase shows generalized periodic discharges with polyspikes (**D**). All these findings improved in the chronic phase (**E**-**H**)

A 60-year-old woman with status epilepticus was admitted to the emergency room. She was an occasional drinker. She had experienced anorexia and gastrointestinal symptoms 3 days previously. On arrival, she was comatose (Glasgow coma scale [GCS] E1VTM1), intubated, not spontaneously breathing, and had tachycardia. She had no fever and her blood pressure was normal. Neurological examination revealed vertical nystagmus and frequent seizures beginning from the face. The seizures stopped following diazepam, fosphenytoin, and midazolam administration. Blood test results revealed acidosis (pH: 7.05) and hyperchloremia (chloride level: 167 mmol/L). Cerebrospinal fluid (CSF) examination revealed no abnormalities. Diffusion-weighted and fluid-attenuated inversion recovery (FLAIR) imaging showed high-intensity lesions in the bilateral medial thalamic nuclei (Fig. 1A, B). Arterial spin labeling (ASL) revealed increased thalamic and markedly decreased cerebral cortical blood flow (Fig. 1C). WE was suspected, and intravenous thiamine administration and steroid pulse therapy (methylprednisolone, 1000 mg/day for 3 days) were initiated. However, the serum thiamine concentration before thiamine administration was found to be normal (27 ng/mL, normal range: 21–81 ng/mL). Electroencephalography (EEG) revealed generalized periodic discharges with polyspikes, similar to a burst suppression pattern (Fig. 1D). Detailed family interviews revealed a history of regular over-the-counter medication use for treating headache. The drug used, NARON ACE T (Taisho Pharmaceutical Co., Ltd. Tokyo, Japan.), contained ibuprofen, ethenzamide, anhydrous caffeine, and BVU. The patient was clinically diagnosed with bromism owing to BVU intoxication, and underwent hemodialysis twice. The serum bromide levels before and after dialysis were 868.2 mg/L and 24.9 mg/L, respectively (normal range: < 10 mg/L). The patient’s consciousness level gradually improved, and she was extubated on day 10 of admission. Although seizures did not recur and the MRI, ASL, and EEG findings improved (Fig. 1E-H), the neurological and psychiatric symptoms persisted, which indicated irreversible brain damage. The patient’s speech was fluent; however, she had phonemic paraphasia, semantic paraphasia, confabulation, delusions, excitability, and anterograde and retrograde amnesia, which was indicative of KS. She was transferred to the psychiatric ward on day 45 of admission.

### Case 2

**Fig. 2:**
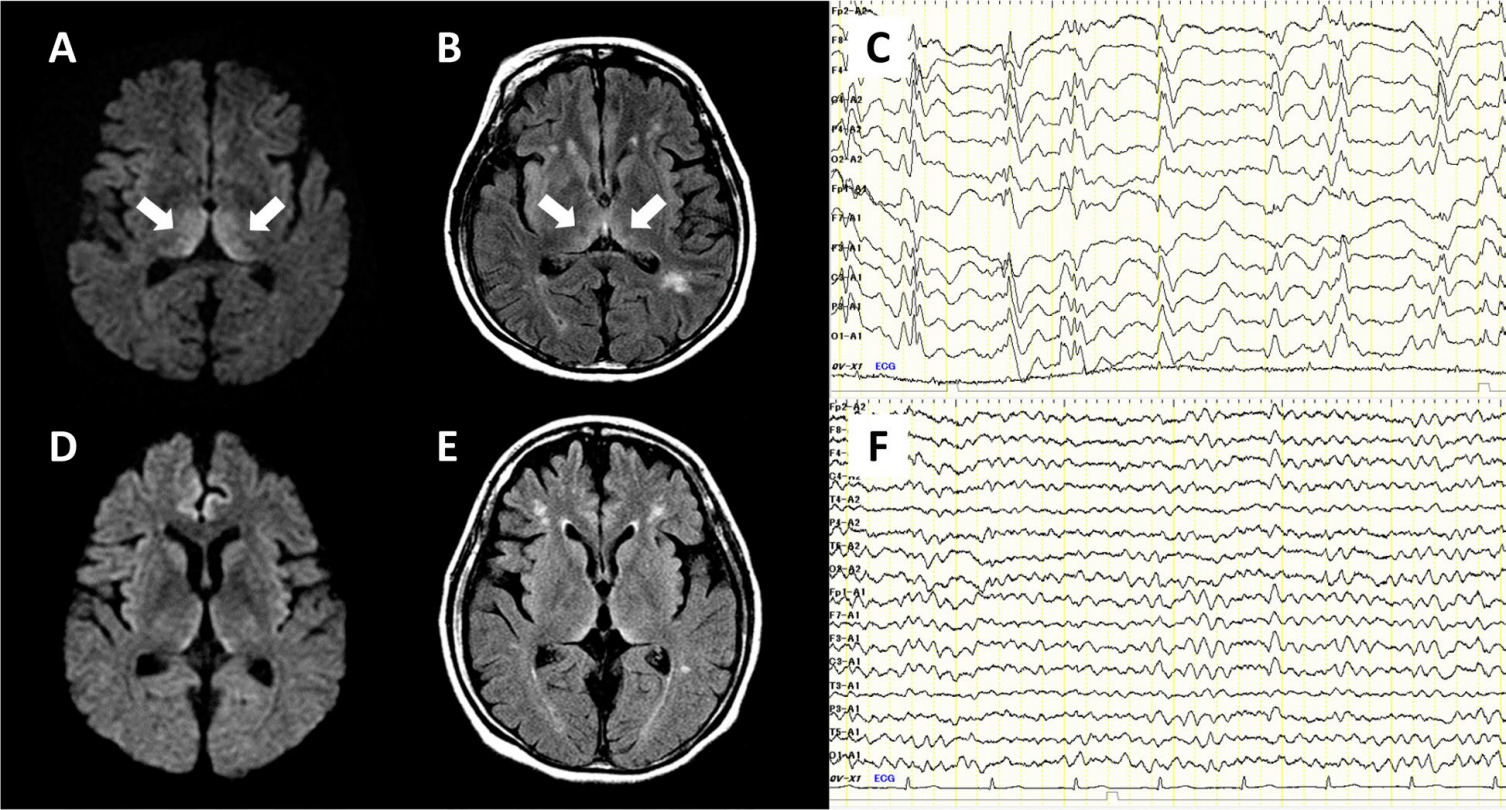
Brain magnetic resonance imaging and electroencephalography findings of Patient 2. Magnetic resonance image (**A**: diffusion-weighted imaging, **B**: fluid-attenuated inversion recovery imaging) obtained in the acute phase shows high-intensity lesions in the medial thalamus (arrows). Electroencephalogram obtained in the acute phase shows generalized periodic discharges with polyspikes (**C**). The findings of Patient 2 are similar to those of Patient 1, and they improved in the chronic phase (**D**-**F**)

A 38-year-old woman presented to the emergency room with status epilepticus. She was an occasional drinker. She had a history of repeated NARON ACE (Taisho Pharmaceutical Co., Ltd. Tokyo, Japan.) overdose. On arrival, she was semi-comatose (GCS E1V1M3) and had tachypnea (respiratory rate: 34/min), shallow breathing, vertical nystagmus, and persisting intermittent facial seizures after a generalized seizure. Initial investigations revealed acidosis (pH: 7.03) and significant hyperchloremia (chloride level: 156 mmol/L). Diffusion-weighted and FLAIR images revealed high-intensity lesions in the bilateral medial thalamic nuclei (Fig. 2A, B). CSF examination revealed no abnormalities. EEG showed periodic polyspikes (Fig. 2C). Intravenous thiamine administration and steroid pulse therapy (methylprednisolone, 1000 mg/day for 3 days) were initiated to treat the suspected WE. However, the serum thiamine concentration before thiamine administration was found to be normal (66 ng/mL). Seizures were treated with phenytoin, levetiracetam, and midazolam. We diagnosed bromism based on the history of regular and excessive NARON ACE use and pseudohyperchloremia, and performed intravenous fluid replacement. The convulsions disappeared within 2 days. The serum and CSF bromide levels were 959 mg/L and 575 mg/L on admission and 151 mg/L and 68 mg/L on day 30 of admission, respectively. The patient’s consciousness level improved from day five of admission and recovered in approximately 1 month. Although MRI and EEG findings improved (Fig. 2D-F), memory and intellectual impairment gradually became apparent, and management in the general ward became difficult. Two months after onset, she was transferred to a closed ward in a psychiatric hospital.

## Discussion and conclusions

### Bromism

The body immediately metabolizes bromine compounds to bromide ions, which are replaced by chloride ions in the extracellular fluid [6]. Bromide reportedly impairs the cell membrane transport in the central nervous system [7], affects the cerebral blood flow [8], damages the cerebellar Purkinje cells, and causes peripheral nerve axonopathy, leading to neurological symptoms, which include disorientation, impaired consciousness, and respiratory depression in acute intoxication, and cerebellar ataxia, dysarthria, and gait disturbance in chronic intoxication. However, since these symptoms can also be caused by pre-metabolized bromine compounds, it may not be appropriate to define them as bromism [9].

History of bromine-compound exposure, pseudohyperchloremia, and characteristic MRI findings are key to diagnosing bromism. Pseudohyperchloremia occurs due to replacement of bromide ions with chloride ions. Additionally, bromide ions are often mistaken for chloride ions on automated analysis using ion-selective electrodes, and bromide ions react more strongly than chloride ions during measurement [10]. Cerebellar atrophy is often noted on MRI in patients with chronic intoxication [11]. In previous reports, symmetrical lesions in the medial thalamus, putamen, periaqueductal gray matter, and cerebellar dentate nucleus have been noted; these lesions were also observed in our patients [1, 12]. These findings were reversible and improved with treatment. Although there is no consensus on the characteristic EEG findings of bromism, there are reports of generalized slow waves, suggesting moderate diffuse cortical dysfunction [1, 4]. In general, burst suppression patterns are seen when cortical activity is suppressed and thalamic cortical neurons fire at high rates as endogenous pacemakers to increase cortical activity [13]. Case 1 is the first instance of bromism evaluation using ASL; the findings of markedly decreased cerebral cortical blood flow and increased thalamic blood flow were consistent with the EEG findings, and may be specific to acute bromism with convulsions. The mainstay of the treatment includes saline loading and diuretic administration. Hemodialysis is effective for treating fulminant bromism and should be performed as early as possible to decrease bromide levels.

In our patients, impaired consciousness persisted and neuropsychiatric sequelae occurred despite prompt reversal of hyperbromemia. Therefore, bromide-induced central nervous system damage should be managed in the hyperacute phase. Furthermore, Patient 1 developed symptoms of acute intoxication despite having no history of overdose with bromide-containing drugs. In individuals with a poor physical condition, poor dietary intake, or marked metabolic acidosis, the blood bromide concentration may increase, inducing sudden-onset acute intoxication. Moreover, the presence of gastrointestinal symptoms before the onset of acute neuropsychiatric symptoms may indicate a state of chronic intoxication. We believe that Patient 1 had preexisting chronic intoxication, and acute intoxication was triggered by temporary malnutrition. The serum chloride levels of patients who regularly use bromide-containing drugs, such as dextromethorphan hydrobromide and BVU, should be regularly checked to prevent the development of acute intoxication and serious sequelae.

### Association with Wernicke–Korsakoff syndrome

Both our patients had imaging findings similar to those associated with WE, a disease caused by thiamine deficiency in which the exact mechanism underlying the neurological symptoms remains unknown. Thiamine deficiency may cause increased glutaric acid levels owing to insufficient energy supply to neurons and decreased enzymatic activity of the citric acid cycle [14], along with changes in the blood-brain barrier (BBB), which is important for maintaining osmotic pressure across the cell membrane [15]. Changes in the BBB and osmotic pressure may be involved in the pathogenesis of bromism. Since chloride ions are involved in the maintenance of osmotic pressure, bromide levels may indirectly affect the osmotic pressure across the cell membrane. This is the mechanism underlying irreversible cortical dysfunction and neuropsychiatric symptoms following acute encephalopathy in both bromism and Wernicke–Korsakoff syndrome. In KS, the lesion sites are thought to be the dorsomedial nucleus of the thalamus and the medial pulvinar, which form the Yakovlev circuit and are circumventricular organs characterized by the lack of a BBB that are involved in maintaining the osmotic pressure across the BBB [16, 17]. This is consistent with the lesion site inferred from imaging findings in our patients. If both bromism and Wernicke–Korsakoff syndrome are caused by osmoregulation abnormalities, they may have common lesion sites. The lack of a BBB may also contribute to the susceptibility to toxic substances in the blood. Patients with WE exhibit hyperintensity in vulnerable areas, such as the medial thalamus on ASL, especially in the acute phase [18]. This is another similarity between the two diseases. In summary, WE is the most important differential diagnosis of bromism because patients with WE present with clinical features of acute encephalopathy and show similar findings on imaging as those with bromism. A detailed medical history and assessment of serum chloride levels are important to differentiate between these two diseases. In our patients, WE was initially suspected based on the imaging findings, but it was ruled out because there was no history of heavy alcohol consumption, chronic malnutrition, or thiamine deficiency. The imaging findings of bilateral thalamic lesions could have indicated other diseases, such as Japanese encephalitis, Leigh syndrome, Creutzfeldt–Jakob disease, and cerebrovascular disease (top of the basilar syndrome) [19]. However, no findings characteristic of any of these diseases were noted in the history or upon examination.

Bromism can cause various neurological symptoms. Patients receiving addictive BVU-containing drugs are susceptible; however, accurately tracking over-the-counter drug use is sometimes challenging. Presence of pseudohyperchloremia and bilateral symmetric thalamic and brainstem lesions on MRI aids diagnosis. Hemodialysis effectively reduces the bromide levels; however, neuropsychiatric sequalae may persist despite early treatment. Further research is needed to understand the pathophysiology and establish effective treatments for the neuropsychiatric sequelae of bromism.

## Data Availability

All data generated or analyzed during this study are included in this published article.

## Abbreviations

BVU: Bromisoval, bromovalerylurea
MRI: Magnetic resonance imaging
WE: Wernicke’s encephalopathy
KS: Korsakoff’s syndrome
GCS: Glasgow coma scale
CSF: Cerebrospinal fluid
FLAIR: Fluid-attenuated inversion recovery
ASL: Arterial spin labeling
EEG: Electroencephalography
BBB: Blood-brain barrier
